# Immunological and Translational Aspects of NK Cell-Based Antitumor Immunotherapies

**DOI:** 10.3389/fimmu.2016.00492

**Published:** 2016-11-11

**Authors:** Maxim Shevtsov, Gabriele Multhoff

**Affiliations:** ^1^Radiation Oncology, Klinikum rechts der Isar, Technische Universität München, Munich, Germany; ^2^Institute of Cytology of the Russian Academy of Sciences (RAS), St. Petersburg, Russia; ^3^Experimental Immune Biology, Institute for innovative Radiotherapy (iRT), Helmholtz Zentrum München, Neuherberg, Germany

**Keywords:** natural killer cell, immunotherapy, monoclonal antibody, antibody-dependent cellular cytotoxicity, checkpoint inhibitors, chimeric antigen receptor

## Abstract

Natural killer (NK) cells play a pivotal role in the first line of defense against cancer. NK cells that are deficient in CD3 and a clonal T cell receptor (TCR) can be subdivided into two major subtypes, CD56^dim^CD16^+^ cytotoxic and CD56^bright^CD16^−^ immunoregulatory NK cells. Cytotoxic NK cells not only directly kill tumor cells without previous stimulation by cytotoxic effector molecules, such as perforin and granzymes or *via* death receptor interactions, but also act as regulatory cells for the immune system by secreting cytokines and chemokines. The aim of this review is to highlight therapeutic strategies utilizing autologous and allogenic NK cells, combinations of NK cells with monoclonal antibodies to induce antibody-dependent cellular cytotoxicity, or immune checkpoint inhibitors. Additionally, we discuss the use of chimeric antigen receptor-engineered NK cells in cancer immunotherapy.

## Introduction

The adoptive transfer of *ex vivo* expanded and/or activated human natural killer (NK) cells represents a promising approach to treat cancer, as NK cells are specialized in the detection and elimination of “modified-self” (1). Apart from T cells, which are capable to recognize tumor-associated foreign antigens (TAA) only when presented on major histocompatibility complex antigen (MHC) molecules through the clonal T cell receptor (TCR), cells of the innate immune system [i.e., NK cells, lymphokine-activated killer (LAK) cells, and cytokine-induced killer (CIK) cells] can recognize and kill neoplastic cells even in the absence of human leukocyte antigen (HLA) and without prior stimulation. NK cells not only control tumor progression but are also engaged in reciprocal interactions with dendritic cells (DCs), macrophages, T cells, and endothelial cells (2). Clinical application of NK cells is an area of intense investigation not only in oncology, especially in hematological malignancies, including leukemia and lymphoma, but also in solid tumors such as ovarian cancer, sarcoma, hepatocellular carcinoma, glioblastoma, and many other types (3–9). Adoptive transfer of autologous or allogeneic NK cells might be superior to the currently widely used donor lymphocyte infusion, which predominantly contain T lymphocytes, due to the fact that NK cells provide the first line of defense and generally mediate less graft-versus-host disease (GvHD) than T cells (10, 11). An alternative for primary NK cells are well-characterized NK-like cell lines such as NK-92, KHYG-1, NKL, and NKG that show antitumor activities (12) and can be easily and reproducibly expanded and applied according to regulatory GMP standards (13, 14).

Based on their tissue distribution and origin, NK cells are divided in bone marrow-derived adult conventional (peripheral) NK cells, thymic-derived, fetal-liver derived, liver resident, uterine-resident intestinal-resident NK cells (15). According to the 14th meeting of the Society of Natural Immunity, it is imperative to harmonize not only the donor source and ultimately donor selection but also the manufacturing and quality control of NK cells used in clinical trials (16). Adult conventional NK cells that are predominantly characterized by the expression of the homomeric adhesion molecule NCAM (CD56) and the low affinity receptor FcyRIII (CD16) and by lacking T cell specific markers such as CD3 and the TCR constitute around 5–20% of peripheral blood lymphocytes. The activity of NK cells is defined by a fine balance of activating and inhibiting receptors belonging to different families including the killer-cell immunoglobulin-like receptors (KIRs), C-type lectin like or natural cytotoxicity class of receptors, and costimulatory receptors (17, 18). According to the surface expression density of CD56 and CD16, NK cells are subdivided into CD56^bright^CD16^−^ (90–95%) that are typically characterized by a low cytotoxicity and a high cytokine production and CD56^dim^CD16^+^ cells (5–10%) with a high cytotoxic activity and a low cytokine release profile (19). CD56^dim^CD16^+^ NK cells that appear first after stem cell transplantation (SCT) or an IL-2-driven *in vivo* therapy are thought to represent a more immature NK cell type (20–22). This subpopulation is hypothesized to change its phenotype and differentiation state throughout its whole lifespan (23) and thus might be of special interest for clinical applications. CD56^bright^CD16^−^ NK cells are considered to exert immunoregulatory functions through the production of Th1 cytokines [i.e., interferon gamma (IFN-γ)] in response to interleukins such as IL-2, IL-12, IL-15, IL-18, and IL-21. They can rapidly proliferate, home to secondary lymphoid organs, and mediate the cross talk between the adaptive and innate immune system (24). In contrast, transforming growth factor-β (TGF-β), IL-10, prostaglandin E2, indolamine 2,3-dioxygenase, adenosine (25), immune checkpoint inhibitors that are produced either by the tumor or its microenvironment as well as immunosuppressive cells such as regulatory T cells (Tregs) and myeloid-derived suppressor cells (MDSCs) can render the NK cell activity silent. Therefore, strategies that antagonize these factors and immunosuppressive cells, the avoidance of tumor hypoxia, the application of immune checkpoint inhibitor antibodies, might be beneficial to overcome the suppression of NK cells.

**Figure 1:**
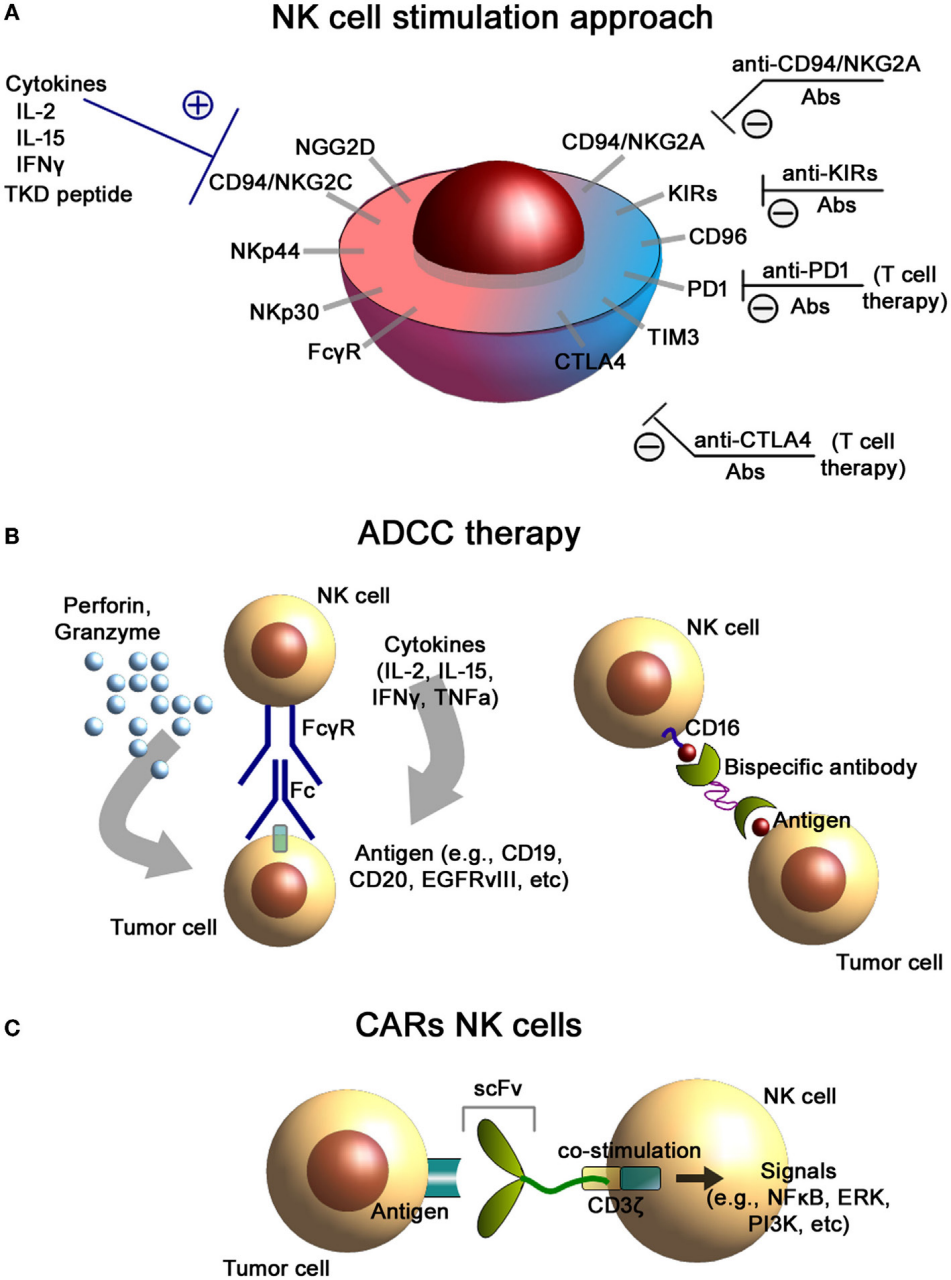
**NK cells-based immunotherapeutic concepts**. **(A)** NK cell stimulation approach. Antibody-mediated blockade of the inhibitory receptors expressed on the cell membrane of NK cells as well as stimulation of the activating receptors results in an increased cytolytic activity of NK cells. **(B)** Antibody-dependent cellular cytotoxicity (ADCC) therapies. Binding of the FcγR to the Fc fragment of the antibody (*left*) results in the activation of NK cells and induces the release of effector molecules such as perforin and granzyme. Application of bispecific antibodies (*right*) directed against CD16 (on NK cells) and tumor antigens facilitate conjugate formation of NK cells with tumor cells. **(C)** Chimeric antigen receptor (CAR)-engineered NK cells. CAR consists of an external recognition domain [i.e., small chain variable fragment (scFv)] that recognizes the tumor-specific antigen, a transmembrane domain, and an intracellular signaling domain (CD3-ζ chain) that induces NK cell activation.

Activation and cytolytic activity of NK cells is dependent upon the activation of NK cell receptors including the natural cytotoxicity receptors (NKp30, NKp44, NKp46), C-type lectin receptors NKG2D, CD94/NKG2C, activatory KIRs, DNAX accessory molecule-1 (DNAM-1, CD226), and costimulatory receptors such as 2B4, NTB-A, CRACC, CD2, CD59, and CD16 (Figure 1A) (26, 27). Additionally, certain cytokines such as IL-2, IL-12, IL-15, IL-18, and IL-21 are known to stimulate both, the proliferative and cytolytic activity of NK cells (28). In order to avoid NK cell-mediated autoimmunity, their cytolytic activities are counterbalanced by the presence of inhibitory receptors such as inhibitory KIRs (22), CD94/NKG2A heterodimers, and checkpoint inhibitor receptors.

## Allogenic and Autologous NK Cell-Based Immunotherapies

### Allogenic NK Cell Approaches

Allogeneic T cells have been shown to be very effective in the treatment of hematological diseases; however, this approach is often hampered by severe GvHD. Therefore, NK cells have been tested in haploidentical SCT settings. In multicenter phase I/II clinical trial, high-risk tumor patients were treated either with freshly isolated or IL-2-stimulated NK-donor lymphocyte infusion (NK-DLI) after haploidentical SCT without any signs of GvHD when less than 25 × 10^3^/kg NK cells were injected (29–31). It was also shown that IL-2-activated NK-DLI was more resistant toward immunosuppressive therapy compared to unstimulated NK-DLI (32), and that these effector cells were able to counteract the immunosuppressive activity of soluble MICA by reactivating NKG2D-mediated cytotoxicity (33, 34). The persistence of *ex vivo* haploidentical IL-2-activated and -expanded NK-DLIs ranges between 7 and 10 days in patients with AML, NHL, and ovarian cancer (35, 36). Allogeneic NK cells that were primed in a two-stage procedure with a leukemic cell line were found to show beneficial effects in patients with resistant AML in a phase I clinical trial (37), and the adoptive transfer of allogeneic CD56^dim^CD16^−^ NK-DLI after haploidentical SCT showed promising results with respect to overall survival in a phase I/II clinical trial with patients with AML, ALL, CML, Hodgkin disease, and MDS (38). Another study using haploidentical KIR-ligand CD56^+^CD3^−^ NK-DLI in elderly AML patients showed an excellent safety profile and promising clinical responses (39).

Allogeneic NK cell therapies after haploidentical SCT show considerable progress within the last decade as determined in phase I/II clinical trials especially in patients with hematological diseases. A better understanding of the nature of NK cell receptors and activities (GvL versus GvHD), improved KIR typing, improved cell purification methods to obtain higher purities, and improved donor selections might improve the clinical outcome of NK cell-based therapies. In addition, further improvements may include novel cytokine cocktails (including IL-12, IL-15, IL-18, and IL-21), the combination of haploidentical NK cell therapies with standard therapies (radiochemotherapy) and/or immune checkpoint inhibitor blockade, and the inclusion of chimeric antigen receptors (CARs) to NK cells. In contrast to hematological diseases, the impact of an NK cell-mediated therapy in solid tumors has to be analyzed in more detailed in further clinical trials.

### Autologous NK Cell Approaches

Due to a large number of immune escape mechanisms of the tumor and its tumor microenvironment, NK cells of tumor patients are frequently tolerant to autologous tumors and have a lower cytotoxic potential compared to NK cells of healthy individuals (40). *Ex vivo* stimulation of NK cells of tumor patients with pro-inflammatory cytokines therefore could be beneficial in enhancing the antitumor immune activity mediated by NK cells. IL-2 (Proleukin) is widely applied and approved in various clinical protocols for the expansion and activation of human effector T and NK cells (40–44), however, with varying success. Furthermore, in cases of a systemic application of high doses of IL-2, severe side effects are induced. A phase I clinical trial indicated that *ex vivo* low dose IL-2 plus Hsp70 peptide-activated, autologous NK cells have been found to be safe and well tolerated even at maximum doses of up to six complete leukapheresis products (45–47). In contrast to IL-2 alone, a stimulation of patient-derived NK cells with Hsp70 peptide plus IL-2 resulted in a reactivation of their cytolytic activity against Hsp70 membrane positive tumor cells (45). The stimulation of NK cells with cluster of differentiation 3 (CD3)-secreted cytokines resulted in a retardation of the growth of melanoma and breast tumors in mice with severe combined immunodeficiency (SCID). In a study by Lamas et al., it was demonstrated that leptin, a hormone–cytokine produced primarily by the adipose tissue, increased NK-92 cell metabolic activity and modulated NK cell cytotoxicity toward cancer cells due to an upregulated TNF-related apoptosis-inducing ligand (TRAIL) and IFN-γ expression (48). Another attractive cytokine candidate that was shown to enhance *in vitro* and *in vivo* NK cell-mediated cytotoxicity and cytokine production is IL-15 (49, 50).

Multimodal therapy approaches in cancer therapy that employs chemotherapy with NK cell-based immunotherapy could also be potentially harmed by drug-mediated cytotoxicity toward NK cells. An interesting approach proposed by Dasgupta et al. showed that genetically engineered NK-92 cells that were resistant to both temozolomide and trimetrexate (51) showed significant therapeutic efficacy in a NOD/SCID/γ-chain knockout (NSG) neuroblastoma mouse model with respect to tumor regression and survival rates in comparison to animals receiving non-engineered cell-based therapy and chemotherapy (51). Presumably, further combinations with other therapeutic approaches can further increase the efficacy of the adoptive transfer of autologous NK cells. Finkel et al. could show significantly enhanced antitumor effects mediated by NK cells following ionizing radiation and hyperthermia treatment. These data provide evidence that a combination of standard therapies such as radiotherapy, hyperthermia, and immunotherapy is feasible (52).

### ADCC-Based Immunotherapy

Natural killer cells play a significant role in facilitating antitumor immunotherapies based on the application of the tumor-targeting monoclonal antibodies. One of the possible immunotherapeutic approaches is based on the antibody-dependent cellular cytotoxicity (ADCC), which is mediated by NK cells (Figure 1B). NK cells are known to express both FcγRIIC/CD32c and FcγRIIIA/CD16a receptors that specifically bind the Fc region of IgG antibodies (53, 54). FcγRIIIA is usually associated with FcRI-γ chains or CD3-ζ chains that are known to have immune tyrosine-based activating motifs (ITAM) in the cytoplasmic domain (55). Following activation of the FcγR, these ITAM are phosphorylated (i.e., activation of signal transduction pathways including PI3K, NFkB, and ERK pathways) that leads to NK cell cytokine secretion and tumor cell lysis (56). ADCC of NK cells is based on (i) secretion of the cytotoxic granules (containing perforin and granzymes), (ii) TNF-mediated signaling, and (iii) pro-inflammatory cytokine release (i.e., IFNγ) (57).

Binding to the Fc fragment leads to the activation of cytotoxicity of NK cells that is utilized in the treatment of various types of cancers characterized by the overexpression of the certain tumor-associated antigens. To date, several ADCC therapies have been assessed in clinical trials including anti-CD20 mAbs (non-Hodgkin’s lymphoma, chronic lymphocytic lymphoma), anti-ganglioside D2 (anti-GD2) mAbs (neuroblastoma, melanoma), anti-human epidermal growth factor (anti-HER2) mAbs (breast and gastric cancers), anti-epidermal growth factor receptor (anti-EGFR) mAbs (colorectal and head and neck cancer), and many other tumor entities (58–62). For the ADCC therapy, various types of antibodies (i.e., mouse antibodies, chimeric antibodies, and fully humanized antibodies) were applied. Thus, Veluchamy et al. (63) demonstrated that supplementary anti-EGFR-targeted therapy using monoclonal antibodies (i.e., cetuximab, panitumumab) significantly enhanced cytotoxic activity of NK cells toward EGFR-positive colorectal tumor cells. Furthermore, cetuximab, a chimeric mAb, was FDA approved for the treatment of EGFR-overexpressing metastatic colorectal cancer, metastatic non-small cell lung cancer, and head and neck cancer (64). The main limitation of the proposed ADCC approach is the source of tumor-specific target antigens. Presumably, combination of mAbs that target various tumor antigens could enhance the efficacy of the therapy. Also, the polymorphism of both FcγRIIC and FcγRIIIA can influence the affinity of the FcγR toward IgG antibodies and as a result influence ADCC (65, 66).

Alternative approaches to increase the specificity and killing activity of NK cells could be based on genetically engineered bispecific (BiKEs) and trispecific killer engagers (TriKEs) (67) (Figure 1B). These designed antibodies facilitate conjugate formation between NK cells and tumor cells. They usually bind both the tumor antigen and the FcγRIIIA/CD16 (68). Recently, a bispecific tetravalent antibody was introduced that targets both CD30 and CD16 for treatment of the relapsed Hodgkin lymphoma patients (69). Designed constructs exhibited superior cytotoxicity compared to conventional antibodies and were independent of the Fc gamma receptor IIIA (FcγRIIIA) allotypes (70).

### Combination of Immune Checkpoint Inhibitor Blockade and NK Cell Therapy

Application of the immune checkpoint inhibitor blockade could provide another approach for improving NK cell-based therapies (Figure 1A). In the study by Guo et al., introduction of the anti-PD1 blocking antibody to the NK cell therapy not only enhanced the *in vitro* activity of the cells (i.e., higher expression of NK activation receptors NKG2D, NKp44, and NKp30) but also augmented the therapeutic potency in the *in vivo* model of multiple myeloma in mice (71). Thus, treatment of autologous NK cells with blocking anti-PD-1 antibodies (pidilizumab) either alone or in combination with rituximab has been found to restore the cell-mediated cytolytic activity of NK cells in patients with multiple myeloma, renal cell carcinoma, and follicular lymphoma (72, 73).

Application of the anti-NKG2A antibodies could represent a novel immunotherapeutic approach in NK cell-based therapy. CD94/NKG2A is an inhibitory receptor that binds HLA-E. Many solid tumors and hemotological malignancies were shown to overexpress the HLA-E that could lead to the inhibition of the cytotoxic activity of NK cells and CD8^+^ cytotoxic T lymphocytes (74–77). Monalizumab (previously known as IPH2201) represents an anti-NKG2A checkpoint inhibitor that is currently tested in clinical trials including head and neck cancers, advanced solid tumors, ovarian cancers, and CLLs.

Another checkpoint inhibitory strategy is based on the application of blocking antibodies toward KIRs, which are activated through the MHC I class molecules present on tumor cells (78, 79). Anti-KIRs monoclonal antibodies (IPH2101) that target KIR2DL-1, -2, and -3 that are specific for HLA-C molecules were investigated in several phase I/II clinical trials (80, 81). Previous preclinical studies clearly confirmed the efficacy of the KIRs blockade on NK cells with respect to an increase in the cytotoxicity of the immune cells toward cancer cells (82, 83). Other immune checkpoint inhibitors [i.e., antibodies toward T cell immunoglobulin and mucin domain 3 (TIM-3), CD96], which are already used for the reactivation of exhausted T cells in melanoma patients, also demonstrated certain effects on NK cells, but the data are controversially discussed with regard to their therapeutic potential (84–86). The role of a TIM-3 blockade especially after cytokine activation of NK cells also show varying results. Further studies are necessary to elucidate the therapeutic role of the blockade of this checkpoint inhibitor, which is expressed on nearly all NK cells. The concomitant blockade of several inhibitors might increase the efficacy of the NK cell-based immunotherapy, although the induced side effects, such as skin rash, mucosa irritation, diarrhea, colitis, hepatotoxixicty, endocrinopasis, and general autoimmunity, should be considered with caution.

## CAR-Engineered NK Cell Therapy

For a specific tumor, targeting the application of genetically engineered CARs for NK cells might provide a promising strategy. A CAR typically consists of an external recognition domain [i.e., small chain variable fragment (scFv)] that recognizes a tumor-specific antigen, a transmembrane domain, and an intracellular signaling domain that mediates NK cell activation. For signaling, usually the ζ chain of the TCR complex CD3 is employed (Figure 1C). At present, there are three generations of CARs in preclinical and clinical settings: (i) CARs with ζ chain of CD3; (ii) combination of CD3-ζ chain with coactivating proteins (e.g., CD28, CD137, and CD134); and (iii) application of CD3-ζ with multiple coactivating domains (87–89). Target cell lines for the development of CARs can be established NK-like cell lines (1) (e.g., NK-92 cells) (14), freshly isolated peripheral blood NK cells (2, 90), or induced pluripotent stem cells (iPSCs) (3) or after subsequent differentiation into mature NK cells (91, 92).

Modified NK cells were shown to significantly enhance antitumor immune responses. Thus, to facilitate selective target cell recognition and enhanced specific cytotoxicity against B-acute lymphoblastic leukemia (B-ALL), the authors transduced the cells with a lentiviral vector encoding a CAR that carry a composite CD28–CD3ζ domain for signaling and a CD19-specific scFv antibody fragment for cell binding (CAR 63.28.z) (93). Treatment with CAR-modified NK cells in a xenograft model resulted in complete and durable molecular remissions of established primary B-ALL (93). To date, several NK-92-based cell lines have been engineered that express a number of different CARs including CD138 or CS1 (anti-multiple myeloma), human epidermal growth factor receptor 2 (HER2) for solid tumors, CD20, CD19 for B cell leukemias, wild-type EGFR and mutant form EGFRvIII for breast cancer patients with brain metastasis, glioblastoma, and ganglioside protein D2 (GD2) for neuroectodermal tumors (3, 94–104).

In summary, many preclinical studies clearly demonstrate therapeutic efficacy of CAR-engineered NK cell-based therapies. However, only few studies compare the efficacy of first-, second-, and third-generation CARs. Another aspect is the choice of the source of NK cells. NK cell lines provide an interesting alternative for peripheral blood-derived NK cells because there is an unlimited availability. However, the density of activatory NK cell receptors on NK cell lines is generally lower, an irradiation prior infusion is necessary, and therefore the *in vivo* persistence is reduced. In contrast, peripheral blood NK cells carry a wider range of activating receptors (i.e., CD16, NKp44, and NKp46) and could be administered without prior irradiation and thus might improve the clinical effectiveness of the cell therapy (105). Another challenge of genetically engineered CARs is the choice of the tumor target. Future clinical applications might utilize several targets to increase the specificity of CAR-based therapies. Modification of the NK cells that makes them insensitive to the immunosuppressive tumor milieu could further increase the efficacy of CAR-NK cells to kill solid tumors that are resistant to NK cell-based immunotherapy.

Alternative approaches are based on the NK cell line NK-92 that is insensitive toward immunosuppressive cytokines (e.g., TGF-β). In a study by Yang et al., it was shown that adoptive transfer of a dominant negative TGF-β type II receptor (DNTβRII) into NK cells significantly decreased tumor proliferation in a lung cancer mouse model (106). Moreover, as recently shown by the group of Genßler et al., genetically engineering NK cells have been shown to overcome tumor heterogeneity by targeting different tumor targets (107). Dual-specific NK cells that recognize both EGFR and EGFRvIII antigens are superior to the monospecific CAR-NK cells in the therapy of glioblastoma (107).

## Conclusion

Natural killer cells-based immunotherapies provide a promising approach that could be employed as an adjuvant anticancer therapy. Most likely, a combination of *ex vivo* cytokine-stimulated autologous or allogenic NK cells with other immunomodulators (i.e., monoclonal antibodies, immune checkpoint inhibitor blockade) and/or standard therapies (i.e., chemo- and radiotherapy) might exert beneficial effects in the treatment of cancer. CAR-engineered NK cells represent a novel immunotherapeutic strategy that could increase the specificity NK cells. Although most NK cell-based therapies are presently in the preclinical phase, recent advances in the therapeutic strategies as well as modifications of the NK cell biology will significantly contribute to the clinical efficacy of NK cell-based therapeutic concepts.

## Author Contributions

MS and GM wrote and approved the final version of the manuscript.

## Conflict of Interest Statement

The authors declare that the research was conducted in the absence of any commercial or financial relationships that could be construed as a potential conflict of interest.
